# Review of intervention products for use in the prevention and control of anemia

**DOI:** 10.1111/nyas.15062

**Published:** 2023-09-08

**Authors:** Daniel Lopez de Romaña, Alison Mildon, Jenna Golan, Maria Elena D. Jefferds, Lisa M. Rogers, Mandana Arabi

**Affiliations:** 1 Global Technical Services, Nutrition International, Ottawa, Ontario, Canada; 2 Nutrition Branch, Centers for Disease Control and Prevention, Atlanta, Georgia, USA; 3 Department of Nutrition and Food Safety, World Health Organization, Geneva, Switzerland

**Keywords:** anemia, interventions, management, prevention

## Abstract

Anemia remains a major public health problem, especially in low- and middle-income countries. The World Health Organization recommends several interventions to prevent and manage anemia in vulnerable population groups, including young children, menstruating adolescent girls and women, and pregnant and postpartum women. Daily iron supplementation reduces the risk of anemia in infants, children, and pregnant women, and intermittent iron supplementation reduces anemia risk in menstruating girls and women. Micronutrient powders reduce the risk of anemia in children. Fortifying wheat flour with iron reduces the risk of anemia in the overall population, whereas the effect of fortifying maize flour and rice is still uncertain. Regarding non-nutrition-related interventions, malaria treatment and deworming have been reported to decrease anemia prevalence. Promising interventions to prevent anemia include vitamin A supplementation, multiple micronutrient supplementation for pregnant women, small-quantity lipid-based supplements, and fortification of salt with iodine and iron. Future research could address the efficacy and safety of different iron supplementation formulations, identify the most bioavailable form of iron for fortification, examine adherence to supplementation regimens and fortification standards, and investigate the effectiveness of integrating micronutrient, helminth, and malaria control programs.

## INTRODUCTION

Anemia continues to be a global public health concern, especially among children, adolescent girls, women of reproductive age, and pregnant and postpartum women in low- and middle-income countries (LMICs). Approximately 40% (269 million) of children 6–59 months of age, 30% (571 million) of women 15–49 years of age, and 37% (32 million) of pregnant women are affected by anemia.^1^ Greater progress is needed to meet the World Health Assembly global nutrition target of a 50% reduction of anemia in women of reproductive age (15–49 years) by 2025; according to current trends, only one LMIC is on track to achieve this target.^2^ The COVID-19 pandemic has further exacerbated food and nutrition insecurity, particularly for women and children in LMICs, resulting in an estimated additional 2.1 million anemic pregnant women in 2020–2022 compared to 2019.^3^ This underlines the need for urgent action to identify and scale proven interventions.

The primary causes of anemia include nutritional deficiencies, inherited red blood cell disorders, infections (e.g., malaria, schistosomiasis, and hookworm) and inflammation, and various conditions (e.g., gynecological disorders in women, gastrointestinal disease, and chronic diseases) that lead to inadequate production of erythrocytes, increased destruction of red blood cells, or blood loss.^4^ Nutritional deficiencies remain an important driver for anemia in children, adolescents, and women globally; iron deficiency is still the most common nutritional deficiency leading to anemia in these population groups, although there is significant heterogeneity in the etiology of anemia, depending on the context.^5–7^

Given its complex etiology, there are multiple interventions relevant to anemia prevention and control. As one of four background papers supporting the *Comprehensive framework for integrated action on the prevention, diagnosis, and management of anemia*,^8^ this paper presents the current World Health Organization (WHO) guidelines and supporting evidence for interventions addressing the major causes of anemia, with a focus on product-based public health interventions. These include micronutrient supplementation and fortification; products for the prevention and treatment of malaria, soil-transmitted helminthiases, and schistosomiasis; and products for the management of blood losses due to postpartum hemorrhage (PPH) and menstruation. Interventions such as dietary counseling that involve service provision without product delivery are not covered in this paper, but the web annex to the *Comprehensive framework* provides an overview of all WHO-recommended interventions.^8^ A brief overview of therapeutic products is included here, as well as the current state of evidence for promising interventions that are not yet recommended by the WHO but hold the potential to contribute to anemia prevention and control. Unless noted otherwise, this paper presents efficacy evidence derived from systematic reviews and meta-analyses, the details of which are provided in Table 1.

## WHO-RECOMMENDED PREVENTIVE INTERVENTIONS FOR ANEMIA

### Supplementation

#### Iron supplementation in preterm and low birth weight infants

For preterm or low birth weight infants receiving human milk and without another source of iron, the WHO strongly recommends daily supplementation with 2–4 mg elemental iron per kg once enteral feeding is established, continuing until another source of iron is introduced (moderate quality evidence).^9^ The meta-analysis informing this recommendation found an increase in hemoglobin (mean difference [MD] = 4.79) and a 75% reduced risk of anemia at follow-up, with no evidence of harm.^10^

#### Iron supplementation in children 6 months to 12 years of age

The WHO recommends that infants and children 6 months to 12 years of age receive daily oral iron supplements for 3 consecutive months, where the prevalence of anemia in infants and young children is 40% or higher.^11^ The recommended dosages of elemental iron are 10–12.5 mg for infants 6–23 months, 30 mg for children 24–59 months, and 30–60 mg for children 5–12 years of age. For all children, except those 24–59 months of age, iron supplementation is recommended for preventing iron deficiency and anemia.^11–13^ For children 24–59 months of age, the recommendation is indicated for increasing hemoglobin concentrations and iron status, not for preventing anemia, given the lack of robust evidence on the effect of daily iron supplementation on anemia for this age group.^11,14^

Children 4–23 months of age who received daily oral iron supplements had close to 40% lower risk of anemia, 70% lower risk of iron deficiency, and 86% lower risk of iron deficiency anemia.^13^ Among 17 studies contributing data for meta-analysis of effects on anemia, 13 provided ≤12.5 mg of iron, primarily as ferrous sulfate. The average intervention duration was 4.3 months (range: 0.25–14 months). Some studies included cointerventions, such as other micronutrient supplements, administered to both intervention and control groups. Subgroup analysis found a 44% lower risk of anemia among studies with iron-only interventions compared with a 37% lower risk among studies with cointerventions.

In children 2–5 years of age, a review of 15 trials providing daily oral iron supplementation found significant increases in hemoglobin and serum ferritin concentrations (MD 6.97 g/dL and MD 11.64 µg/L, respectively), but only one study assessed effects on anemia, precluding meta-analysis.^14^ Nine studies provided iron as ferrous sulfate, with the dose ranging from 10 to 65 mg/day; three did not specify the formulation, and the remaining three used ferrous fumarate, ferrous gluconate, or ferric ammonium citrate. Intervention duration ranged from 28 days to 12 months.^14^ Subgroup analyses showed meaningful increases in hemoglobin from interventions lasting 1–3 months with total iron intake ≤2500 mg elemental iron, with no evidence of additional benefits from longer interventions or higher iron intake. The effect on hemoglobin was greater among children with anemia or iron deficiency at baseline.

Daily iron supplementation in children 5–12 years of age reduced the risk of anemia by 50% and the risk of iron deficiency by almost 80%.^12^ The intervention was also associated with significant increases in serum ferritin and hemoglobin concentrations. Most studies (19/32) provided iron supplementation as ferrous sulfate, with doses ranging from 5 to 120 mg/day. Among studies contributing data to the meta-analysis of effects on anemia, six out of seven provided 31–60 mg elemental iron. The duration of supplementation ranged from 1 to 12 months.^12^

A recent meta-review of systematic reviews corroborates that daily iron supplementation increases hemoglobin concentration and decreases the risk of anemia by 41% in children 6–59 months of age. Improved hemoglobin concentration was also reported for children ≥5 years of age, but this review did not include trials assessing the impact of daily iron supplementation on anemia in this age group.^15^

#### Iron supplementation for children in malaria-endemic areas

The WHO recommends that oral iron supplements not be given to infants and children in malaria-endemic areas without access to public health measures to prevent, diagnose, and treat malaria.^11^ A 2006 landmark study on Pemba Island, Tanzania, a malaria-endemic area, found that universal daily supplementation with iron and folic acid (IFA) compared with placebo significantly increased the risk of hospitalization and death in young children; a substudy involving more intensive infection management found reduced risk of adverse events among supplemented children with baseline iron deficiency and anemia.^16^ Three subsequent Cochrane Reviews found no harmful effects of iron supplementation in children residing in malaria-endemic areas if malaria control and treatment resources were in place.^17–19^

#### Iron supplementation for menstruating women and adolescent girls

The WHO recommends daily supplementation with 30–60 mg elemental iron for 3 consecutive months each year for nonpregnant, menstruating women and adolescent girls in settings where anemia prevalence in this population group is 40% or higher.^20^ Where anemia prevalence is 20%–39%, or when daily supplementation is not tolerated, the WHO recommends weekly supplementation with 60 mg elemental iron (and 2.8 mg folic acid) in a repeated rotating schedule of 3 months of supplementation followed by 3 months without.^21^

Daily iron supplementation reduced the risk of anemia by 60% and iron deficiency by almost 40% in menstruating adult women and adolescent girls.^22^ Hemoglobin concentration and exercise performance also appeared to improve with the intervention. The dose of iron provided ranged from 3 to 420 mg/day, and the duration ranged from 7 days to 6 months. The limited available data suggest that ferrous sulfate may be more effective than other iron formulations for reducing anemia.^22^ Intervention dose and duration do not appear to influence the effects on anemia. In some studies, iron was coadministered with folate and/or vitamin C, but the efficacy of iron alone compared with cointerventions has not been determined due to limited data availability. Evidence of adverse effects has been assessed differently across studies, with some reporting no effects and some reporting gastrointestinal side effects, with high-quality evidence for both loose stools/diarrhea and hard stool/constipation. No studies have reported the effect of daily iron supplementation on mortality in menstruating adult women and adolescent girls.^22^

Intermittent oral iron supplementation (once or twice weekly) has similar effects to daily regimens among menstruating girls and women, reducing the risk of anemia by 35% and with almost 60% lower risk of adverse effects.^23^ Effectiveness did not vary based on dosage (greater or less than 60 mg elemental iron per week), frequency of supplementation (once or twice weekly), intervention duration (longer or shorter than 3 months), or malaria endemicity. Morbidity outcomes, including malaria, have not yet been adequately assessed.

#### Iron or IFA supplementation for pregnant women and adolescent girls

The WHO recommends daily supplementation with 30–60 mg of elemental iron and 0.4 mg folic acid during pregnancy, beginning as early as possible, with total consumption of at least 180 tablets.^24^ Daily supplementation with iron or IFA has been found to decrease the risk of maternal anemia by 60%–70% and the risk of iron deficiency by 57% but does not appear to influence severe anemia in the second or third trimester of pregnancy, although evidence quality is low.^25^ Pregnant women who receive daily iron or IFA supplements are likely to have higher hemoglobin levels at term and 6 weeks postpartum. However, they may also be at increased risk of hemoglobin concentrations greater than 130 g/L during pregnancy and at term, which is associated with a higher risk of adverse pregnancy outcomes.^25^ These results should be interpreted with caution due to high heterogeneity across studies. Daily iron or IFA supplementation does not appear to influence other maternal outcomes, including mortality, reported side effects, severe anemia or infection during pregnancy, or infant outcomes.^26^ Among 14 studies contributing data on anemia, two studies provided daily doses of <30 mg of elemental iron, three studies provided 30–60 mg, five studies provided 61–120 mg, two studies provided 200 mg, one study provided 900 mg, and one study did not disclose the amount of elemental iron provided. Only two studies provided IFA, with folic acid doses of 0.5, 1, and 2 mg. In most of the trials, women began taking supplements before 20 weeks of gestation and continued taking supplements up until delivery.^26^

#### Intermittent IFA supplementation for pregnant women

The WHO recommends weekly supplementation with 120 mg elemental iron and 2.8 mg folic acid for pregnant women when daily IFA is not acceptable due to side effects, or in populations with anemia prevalence below 20% among pregnant women.^24^ When intermittent supplementation is compared to daily supplementation, there is no significant difference in maternal anemia or iron-deficiency anemia at term, but the quality of the evidence is very low.^25^ Intermittent IFA supplementation could reduce the risk of high hemoglobin levels in mid and late pregnancy but may increase the risk of mild anemia near term. However, women receiving intermittent iron supplements are less likely to report side effects than those receiving daily supplements. Most trials used intermittent regimens where women took supplements 1 day each week. The dose of iron ranged from 80 to 300 mg of elemental iron per week.^25^

#### Iron supplementation for pregnant women in malaria-endemic areas

The WHO recommendation for daily antenatal iron supplementation is universal, but concurrent management of malaria is recommended in endemic areas.^24^ Subgroup analysis of iron or IFA supplementation trials found beneficial effects on anemia in both malarial and nonmalarial settings.^25^ A narrative literature review including both randomized trials and observational studies concluded that epidemiological evidence does not indicate an increased risk of *Plasmodium falciparum* infection with antenatal iron supplementation at WHO-recommended levels.^27^

#### Iron or IFA supplementation for postpartum women

The WHO conditionally recommends the provision of iron or IFA supplements to postpartum women for 6–12 weeks following delivery to reduce the risk of anemia in settings where the prevalence of gestational anemia is 20% or greater (low-quality evidence).^28^ This is a conditional recommendation—indicating that there is a need for substantial debate and involvement from stakeholders before considering the adoption of the recommendation—due to the limited data on this intervention.

#### Iron compounds for supplementation

Ferrous iron salts are commonly used for oral iron supplementation, particularly ferrous sulfate, as it is low-cost and well-tolerated.^29^ Iron bioavailability from ferrous salts is around 10%–15%. This is markedly higher than the bioavailability from ferric iron preparations (such as iron polymaltose complex), which must be transformed into ferrous iron for absorption.^29^ Fractional iron absorption from oral supplements is also influenced by concurrent food consumption, and by the increase in hepcidin (the primary peptide regulating physiologic iron homeostasis) following intake of >100 or >60 mg iron in anemic and nonanemic women with iron deficiency, respectively.^30^ Consumption of iron supplements on alternate rather than consecutive days has been shown to improve fractional iron absorption.^31^ Iron supplements are associated with gastrointestinal side effects, which can be mitigated in women with doses ≤50 mg iron per day,^32^ or by using slow-release compounds,^33^ although these are not widely available in public health programs.

Oral iron supplements typically provide a relatively high dose of iron as a ferrous salt to ensure sufficient iron absorption in the context of diets high in iron inhibitors and increased hepcidin levels due to inflammation. However, these higher doses incur an increased risk of gastrointestinal inflammation and diarrhea, which is likely due to alterations to the microbiome in response to excess colonic iron that may decrease gut *Bifidobacteriaceae* and *Lactobacillaceae* and increase enteropathogens and diarrhea.^34^ Promising approaches to mitigate this adverse effect include the consumption of prebiotics alongside iron supplements, which can selectively enhance the growth of *Bifidobacteriaceae* and *Lactobacillaceae*, or the replacement of ferrous salts with sodium iron (III) ethylenediaminetetraacetate (NaFeEDTA).^34^ NaFeEDTA is a highly soluble chelating agent with higher iron bioavailability than ferrous salts, but the safe upper limit of consumption is based on body weight, so only low doses can be used in products for young children.^35^ Consumption from all sources should not exceed 1.9 mg EDTA/kg/day.^36^

### Point-of-use fortification of foods with multiple micronutrient powders

The WHO strongly recommends point-of-use fortification of complementary foods with iron-containing micronutrient powders (MNPs) for infants and young children 6–23 months of age, and children 2–12 years to improve iron status and reduce anemia.^37^

#### MNPs for children 6–23 months of age

In children 6–23 months of age, MNPs decrease the risk of anemia by 18% and iron deficiency by 53% compared to no intervention or placebo.^38^ MNPs appear to improve some cognitive outcomes, such as receptive language (MD = 0.17) and expressive language (MD = 0.13), but do not seem to influence all-cause morbidity, diarrhea, upper respiratory infections, or malaria. Mortality and iron overload have not been reported.^38^ MNPs appear to be more effective for children who are anemic in nonmalarial settings when provided daily and when the intervention lasts less than 6 months. The formulation of MNPs varied across studies, and the number of micronutrients included ranged from 5 to 22. Most studies provided either 10 or 12.5 mg of elemental iron as ferrous fumarate, with iron content below 12.5 mg found to be less effective. All formulations included zinc, vitamin A, and folic acid. Most studies provided MNPs daily, with 10/23 doing so for <6 months and 13/23 for 6 months or more.^38^

#### MNPs for children 2–12 years of age

MNPs were found to reduce the risk of anemia and iron deficiency in children 2–12 years of age by 34% and 65%, respectively.^39^ It appears that MNPs have no impact on diarrhea or other adverse side effects in this age group.^39^ MNP composition, including iron compound and dose, varied across studies, with 10 trials providing less than 12.5 mg of iron. All included daily provision of MNPs in at least one arm of the study. The duration of the intervention ranged from 2 to 12 months.^39^

### Staple food fortification

#### Wheat flour

The WHO strongly recommends fortifying wheat flour with highly bioavailable iron as a public health strategy to improve hemoglobin concentration and iron status and to prevent anemia and iron deficiency.^40^ Fortification of wheat flour with iron, with or without other micronutrients, reduced the risk of anemia by 27% in the general population above 2 years of age, although effects on iron deficiency and hemoglobin concentrations are less certain.^41^ The baseline prevalence of anemia in the population (20%–39% vs. ≥40%), amount of elemental iron added to the flour (<40 vs. >60 mg/kg), type of iron compound (high vs. low bioavailability), or duration of the intervention (<6 months vs. 6–12 months) did not influence the result. Studies did not report on adverse effects, other than two studies showing no overall effect on inflammation or infection in children 2–11 years of age as measured by C-reactive protein (CRP) (MD = 0.04). The doses of iron used, types of iron salt provided, and duration of the interventions were heterogeneous across studies. The level of iron fortification ranged from 10 mg to 200 mg/kg of wheat flour, with most trials using ≥40 mg/kg. The duration for most studies ranged from 3 to 8 months, with one study lasting 24 months.^41^ It is uncertain whether fortification of wheat flour with iron in combination with other micronutrients decreases the risk for anemia, mostly due to the scarcity of studies and the risk of bias of these studies.^41^

#### Maize flour

The WHO recommends fortification of maize flour and corn meal with iron to prevent iron deficiency in populations, but this is based on very low-quality evidence.^42^ The WHO noted the positive effects on the nutritional status of using fortified foods in general to supply sufficient amounts of micronutrients that are otherwise inadequate in the diet, compared to no intervention. However, it is uncertain whether fortified maize flour decreases the risk of anemia, iron deficiency, or iron-deficiency anemia as robust evidence is still lacking.^43^ Studies included children aged 2–11 years and women. The maize flour used varied (nixtamalized maize flour, whole milled maize, and degermed), and the duration of studies ranged from 6 to 10 months. All studies compared maize flour fortified with iron and other micronutrients versus unfortified maize flour, but iron and other fortificant formulations varied and doses of iron ranged from 28 to 140 mg/kg.^43^ None of the studies reported on adverse effects.

#### Rice

The WHO recommends fortifying rice with iron and vitamin A to improve the iron status of populations in settings where rice is a staple food, but robust evidence of effects on hemoglobin and anemia is not yet available.^44^

A 2019 review found that rice fortified with iron alone or with iron plus other micronutrients may reduce anemia by 28%, iron deficiency by 44%, and increase hemoglobin concentration by 1.83 g/L, but the quality of evidence is low.^45^ Micronutrient content (iron alone vs. iron plus other micronutrients), extrusion method (hot vs. cold), anemia at baseline (5%–39% vs. ≥40%), and malaria endemicity (malaria-risk area vs. malaria-free area) did not seem to influence the effect of rice fortification on anemia. The inclusion of vitamin A in the micronutrient premix did not appear to affect vitamin A status but may improve hemoglobin (MD = 10.0); evidence quality is low for both these findings. Evidence of adverse effects was limited. One study found that fortified rice increased the risk of hookworm infection, and another found no impact on abdominal pain lasting more than 3 days.^45^ Studies were conducted among nonpregnant, nonlactating women 18–49 years of age and among preschool and school-age children. Studies assessed both hot- and cold-extrusion methods, with 8/17 studies using hot extrusion. All studies fortified rice with iron and some studies included other micronutrients. Iron compounds included ferric phosphate tetrahydrate, ferrous sulfate, and ferric pyrophosphate. The amount of elemental iron ranged from 20 to 1128 mg/kg of rice. The manner of cooking rice was not described in sufficient detail.^45^

#### Fortification of foods with multiple micronutrients

In addition to the WHO’s universal fortification recommendations reviewed above, global guidelines on food fortification are applicable to a wide variety of food vehicles.^29^ Fortification of staple foods with multiple micronutrients decreases the risk of anemia by 32%, iron deficiency anemia by 72%, and iron deficiency by 56% and increases hemoglobin by 3 g/L and serum ferritin concentrations by 8.3 µg/mL.^46^ Food vehicles assessed included rice, wheat flour, dairy products, nondairy beverages, biscuits, spreads, and salt.

#### Iron compounds for food fortification

In cereal staple food fortification, the bioavailability of micronutrient fortificants depends in part on the grain type and extraction rate of the flour during milling, which determine phytate levels. Water-soluble iron compounds (e.g., ferrous sulfate) are more bioavailable than compounds that are soluble in dilute acids (e.g., ferrous fumarate), whereas water-insoluble compounds are the least bioavailable (e.g., reduced iron or ferric pyrophosphate).^29^ NaFeEDTA is recommended for fortifying wheat flour at both low and high extraction rates, while ferrous sulfate and ferrous fumarate are only recommended for low extraction rates, and electrolytic iron is recommended for low extraction rates when average daily per capita consumption of fortified wheat flour is estimated to be at least 150 g.^40^ Fortification costs are lowest with electrolytic iron, increase with ferrous sulfate, and are substantially higher with NaFeEDTA.^47^ Considerations related to bioavailability, safety, efficacy, and cost must be balanced with effects on the sensory properties of the food, which is a major influence on the acceptability of the fortified product. Further research is needed on the bioavailability of different iron formulations added singly and in combination with other micronutrients, the effects of different phytate levels on the absorption of iron fortificants, and the stability of micronutrients during context-specific cooking processes.^40^

### Malaria prevention

#### Malaria chemoprevention for children under 2 years of age

The WHO recommends perennial malaria chemoprevention (PMC) for children under 2 years of age in areas of moderate to high perennial malaria transmission, although there are limited available data on the safety and efficacy of intermittent preventative treatment (IPT) in children ≤15 months of age.^48^ PMC using sulfadoxine-pyrimethamine (SP) likely decreases the risk of anemia by 18% (moderate-certainty evidence) but does not appear to increase hemoglobin concentrations.^49^ Studies have evaluated a range of 3–6 doses of SP in infants under 12 months of age and 1–11 doses of SP in infants 12–23 months of age.^50^ There is very limited evidence regarding the effectiveness of other antimalaria medicines for infants, but using mefloquine, amodiaquine-artesunate, or SP-artesunate probably has little or no effect on anemia (moderate-certainty evidence).^49^

#### Malaria chemoprevention for school-aged children

The WHO also recommends IPT for school-aged children living in malaria-endemic settings with moderate to high perennial or seasonal transmission of malaria to reduce disease burden.^48^ IPT may decrease the risk of anemia in school-aged children 5–15 years of age by 15% (low-certainty evidence).^48^

In malaria-endemic areas, the WHO strongly recommends seasonal antimalarial chemoprevention (SMC), the provision of IPT during the transmission season to children in age groups at high risk of severe malaria, regardless of infection status.^48^ SMC likely reduces the prevalence of anemia in children under 5 years of age by 16% (moderate-certainty evidence).^48^ SMC reduces the prevalence of anemia in children 5–9 years of age by 30%,^48^ although this is based on the results of one large study conducted in Senegal.^51^ SMC is also associated with 1.4 times the risk of mild to moderate adverse events—vomiting, abdominal pain, and nausea—in children up to 15 years of age.^48^

The WHO also recommends postdischarge malaria chemoprevention to children admitted to hospital with severe anemia and living in settings with moderate to high malaria transmission, regardless of whether the cause of severe anemia is identified.^48^ This intervention probably reduces the risk for readmission to the hospital for severe anemia by 26% (moderate-certainty evidence).^48^

#### Malaria chemoprevention for pregnant women

The WHO strongly recommends IPT for malaria during pregnancy to reduce disease burden and adverse pregnancy and birth outcomes.^48^ IPT with SP may reduce the risk of anemia in pregnant women by 10% and may increase hemoglobin by 1.9 g/L.^48^ High doses of folic acid (daily dose ≥ 5 mg) have been shown to counteract the efficacy of SP as an antimalarial, and only low-dose formulations of folic acid (i.e., 0.4 mg daily) should be coadministered with SP.^52^

#### Insecticide-treated bed nets and indoor residual spraying

In addition to IPT, the WHO strongly recommends using insecticide-treated bed nets (ITNs) and indoor residual spraying in areas with ongoing malaria transmission.^48^ ITNs have been shown to increase hemoglobin (expressed as mean packed cell volume) by 1.29 compared to no use of nets (high-certainty evidence), but effects on anemia have not been reported.^53^

#### Management of malaria in the context of glucose-6-phosphate dehydrogenase deficiency

Individuals with glucose-6-phosphate dehydrogenase (G6PD) deficiency, an inherited red blood cell disorder that is widespread in the tropics and subtropics, can develop acute hemolytic anemia if exposed to 8-amiloquinones, which are commonly used to treat malaria due to *P. falciparum* and to treat relapse of malaria due to *P. vivax* and *P. ovale*.^54^ Widespread screening for the presence of G6PD deficiency is not available in most countries, although rapid diagnostic tests for G6PD are in development.^55^ To prevent relapse, the WHO recommends treating infection with *P. vivax* or *P. ovale* in children and adults (except pregnant women, infants aged <6 months, women breastfeeding infants aged <6 months, women breastfeeding older infants unless they are known not to be G6PD deficient, and people with G6PD deficiency) with a 14-day course of primaquine at 0.25–0.5 mg/kg body weight daily in all transmission settings. For those with a diagnosis of G6PD deficiency, relapse can be prevented by providing primaquine at 0.75 mg/kg body weight once a week for 8 weeks, with close medical supervision for potential primaquine-induced hemolysis.^48^

### Deworming

#### Deworming for children and nonpregnant adolescents and adults

The WHO strongly recommends preventative chemotherapy (deworming) using annual or biannual single-dose albendazole or mebendazole for all children 12 months to 12/14 years of age, nonpregnant adolescents, and nonpregnant women of reproductive age living in areas where the baseline prevalence of infection is 20% or higher, to reduce the burden of soil-transmitted helminths.^56^ Although mass deworming campaigns in children seem to be effective in reducing worm burden,^57^ this has not translated into a positive effect on hemoglobin concentrations, either when single or multiple doses of anthelmintics are administered (moderate-certainty evidence).^57,58^ Similar effects of anthelmintics on worm burden and anemia or iron deficiency anemia have been found in adolescent girls and women of reproductive age living in areas that are endemic for soil-transmitted helminths (low-certainty evidence).^59^

A more recent systematic review of preventive chemotherapy in nonpregnant populations (preschool-aged children, school-aged children, adult males, and nonpregnant adult females) found that preventive chemotherapy increases hemoglobin concentrations, although the impact varied by medication, with albendazole increasing hemoglobin by 3.02 g/L and mebendazole not having an effect.^60^ Coadministration of albendazole with praziquantel or with iron supplements did not appear to enhance the effect on hemoglobin.

#### Deworming for pregnant women

The WHO recommends preventive chemotherapy using single-dose albendazole (400 mg) or mebendazole (500 mg) for pregnant women after the first trimester to reduce the worm burden of soil-transmitted helminth infections. This recommendation applies to areas where both the baseline prevalence of hookworm and/or *T. trichiura* infection among pregnant women is 20% or higher, and the prevalence of anemia among pregnant women is 40% or higher.^56^

Evidence for the reduction in worm burden after a single dose of preventive chemotherapy appears to vary by the type of helminth infestation (uncertain for hookworm, low for *Trichuris*, and moderate for *Ascaris*).^61^ This variation may nuance the effect anthelmintics have on anemia, with low-certainty evidence showing that a single dose of anthelmintics (for any type of helminth) in the second trimester of pregnancy may decrease maternal anemia in the third trimester by 15%.^61^ This is an update to the evidence that informed the most recent WHO guidelines (2017) on preventive chemotherapy to control soil-transmitted helminth infections as a public health intervention, which did not find a significant effect of deworming on maternal anemia measured during the third trimester of pregnancy.^56^

### Prevention and treatment of schistosomiasis

The WHO strongly recommends chemoprevention for schistosomiasis, through annual administration of a single dose of praziquantal in order to reduce morbidity and contribute to disease elimination in endemic areas with at least 10% infection prevalence (moderate-certainty evidence).^62^ Treatment with praziquantal is also recommended for infected individuals. These recommendations apply to all individuals above 2 years of age except women in the first trimester of pregnancy. For children below 2 years of age, treatment based on individual clinical assessment is recommended, and a pediatric formulation is in development.^62^ A systematic review of deworming interventions in nonpregnant populations reported an increase in hemoglobin with coadministration of albendazole and praziquantal (standardized mean difference [SMD] = 0.27; 95% CI: [0.18, 0.36]; three studies), but effects of praziquantal alone were not assessed.^60^ Meta-analysis of two studies of preventive chemotherapy for schistosomiasis in school-aged children found a 30% reduction in anemia, but this outcome has not been assessed for other age groups.^62^ There is a need for further evidence regarding the effects of schistosomiasis treatment on anemia.

### Prevention and treatment of PPH with uterotonics

The WHO recommends the use of uterotonics, preferably oxytocin, for both the prevention and treatment of PPH.^63,64^ For prevention of PPH, all women giving birth should be offered oxytocin, administered either intramuscularly or intravenously, during the third stage of labor. Similarly, intravenous oxytocin is the recommended uterotonic drug for the treatment of PPH. Oxytocin probably does not have an effect on the risk of severe anemia (hemoglobin <70 g/L; moderate-quality evidence).^65^

### Prevention and management of menstrual blood loss with hormonal contraception

Hormonal contraceptives are recommended by the WHO for family planning^66^ but may also help mitigate anemia by reducing menstrual blood losses. A Cochrane review found that combined oral contraceptive pills (COCPs) compared with placebo reduce menstrual blood loss and improve hemoglobin levels (moderate-quality evidence), and that the levonorgestrel-releasing intrauterine system is more effective than COCPs at reducing menstrual blood loss (low-quality evidence).^67^

### Promising preventive interventions

The interventions presented above are recommended by the WHO for the prevention of anemia and its direct causes in populations. There is also a growing evidence base for additional product-based interventions with the potential to contribute to anemia prevention and control. This section presents the current evidence for vitamin A supplementation, multiple micronutrient supplementation, lipid-based nutrient supplements (LNSs), the fortification of salt with iron and other micronutrients, and biofortified crops.

#### Vitamin A supplementation

Vitamin A supplementation may protect against anemia in several population groups.^68^ In children and adolescents, vitamin A supplementation reduced the risk of anemia by 28% and increased hemoglobin concentrations by 3.42 g/L.^69^ The dose of vitamin A ranged from 500 to 200,000 IU, with most studies providing a single oral dose. These studies did not report outcomes related to cognitive development or adverse effects.^69^ The current WHO vitamin A supplementation guidelines for children focus on addressing vitamin A deficiency and related health outcomes other than anemia.^70^

In settings where vitamin A deficiency is common, supplementation during pregnancy may decrease the risk of maternal anemia by 35% but does not seem to influence neonatal anemia when compared to a control.^71^ Supplementation with vitamin A in combination with other micronutrients does not reduce maternal or neonatal anemia compared with micronutrient supplementation excluding vitamin A.^71^ Available studies were conducted largely in populations of pregnant women considered to be moderately vitamin A–deficient and most provided between 3000 and 23,300 IU of supplemental vitamin A for 8–12 weeks.^71^ In populations with adequate vitamin A intake, there are no known benefits and an increased risk of adverse effects from antenatal vitamin A supplementation. The WHO, therefore, recommends vitamin A supplementation at doses up to 10,000 IU daily or 25,000 IU weekly for pregnant women only in areas where vitamin A deficiency is a severe public health problem, in order to prevent night blindness while avoiding potential teratogenic effects of high-dose supplementation.^70^

#### Multiple micronutrient supplementation for pregnant women and adolescent girls

The use of daily multiple micronutrient supplements (MMSs) may have the advantage of addressing multiple deficiencies simultaneously; however, the WHO currently does not recommend switching from the use of IFA to MMSs as part of routine antenatal care, except within the context of research.^70^

When comparing the effects of MMSs containing 13–15 micronutrients, including IFA, with IFA only, no differences were found in maternal anemia in the third trimester but MMSs had beneficial effects on infant outcomes.^70^

#### Lipid-based nutrient supplements

Lipid-based nutrient supplements (LNSs) are food supplements (spread/paste) containing lipids as the main energy source and providing protein and micronutrients. LNSs are commonly made with heat-treated peanuts/pulses/cereals, milk powder, vegetable oils, sugar, maltodextrin, and micronutrients. Small-quantity (SQ; ∼20 g) LNS is intended to complement the diet of children 6 months of age and older with essential nutrients and contributes to the prevention of undernutrition. Medium-quantity (MQ; ∼50 g) LNS is intended to prevent malnutrition in children 6 months of age and older. Large-quantity (∼500 kcal) LNS is often referred to as a ready-to-use supplementary food and used for the treatment of moderate acute malnutrition.

Either SQ- or MQ-LNS plus complementary feeding may increase hemoglobin concentration (MD = 5.78 g/L) and reduce the risk of anemia by 21% in children 6–23 months of age, compared with no intervention (low certainty of evidence).^72^ Compared to MNP, SQ-LNS was found to increase hemoglobin (MD = 5.13) and reduce the risk of anemia by 62% based on two studies (low-quality evidence), but findings were no longer significant when one study at high risk of bias was removed from the analysis.^72^ Adverse effects did not differ between children receiving LNS plus complementary feeding and those not receiving an intervention, suggesting that LNS plus complementary feeding is safe for this age group.^72^ LNS formulation was heterogeneous across studies, but all included iron, with the dose of elemental iron varying between 6 and 9.5 mg. Most studies provided LNS at 6 months of age with the duration of the intervention ranging from 3 to 18 months.^72^

A recent individual participant data meta-analysis of 14 randomized controlled trials of SQ-LNS provided to children 6–23 months found that children receiving LNS had a 16% lower prevalence ofanemia, 56% lower prevalence of iron deficiency, and 64% lower prevalence of iron-deficiency anemia compared with controls, with greater improvements in hemoglobin status in populations with a high prevalence ofanemia.^73^

Only one study has compared LNS during pregnancy to IFA or MMS. In both comparisons, participants receiving LNS were more likely to develop maternal anemia at term or near term.^74^

#### Double-fortified salt with iron and iodine

Salt iodization is a successful strategy for controlling iodine deficiency disorders. The WHO currently discourages the use of salt as a vehicle for fortification with nutrients other than iodine and fluoride.^75^ However, trials have shown that the provision of salt fortified with both iodine and iron (double-fortified salt; DFS) may reduce the risk of anemia by 20%–40% and the risk for iron deficiency anemia by approximately 65% in the general population.^76–78^ DFS may increase hemoglobin and serum ferritin concentrations but evidence to date is of low quality and studies have not reported on adverse side effects. Micronized ferric pyrophosphate may produce greater reductions in the risk of anemia compared to ferrous sulfate and ferrous fumarate.^77,78^ Most studies were conducted in school-age children and/or women of reproductive age, lasted between 6 and 12 months, and provided 1 mg/g of salt.^77,78^ Due to production costs, the use of DFS could be most feasible in high-income countries, potentially reducing the impact of anemia in low socioeconomic strata in those settings.^79^

#### Biofortification of crops

Biofortification is the process by which the nutritional content of staple crops is changed through various agronomic technologies, including the application of micronutrient fertilizer to crops, conventional plant breeding, and/or genetic modification. Consumption of iron-biofortified crops, such as rice, pearl millet, and beans, does not appear to decrease the risk of anemia or iron deficiency in people aged 12–45 years, but the evidence to date is very limited.^80^ Iron-biofortified crops have the potential to improve cognitive performance with respect to attention and memory domains in vulnerable populations, but available studies have not assessed the effect on mortality or other adverse side effects.^80,81^

### Interventions to treat anemia

This paper and the WHO’s guidance document on the appropriate nutrition actions to prevent and control nutritional anemia^82^ focus on public health approaches to the prevention and control of anemia, but treatment of affected individuals is also essential. Anemia is diagnosed by hemoglobin concentration below established age- and sex-specific cutoffs, but further investigation is needed to identify the underlying cause(s) that are the focus of treatment.^4^ Primary treatment for common causes of anemia is briefly presented in this section, with further details available in relevant clinical protocols.

For anemia due to absolute iron deficiency, treatment with oral iron salts (sulfate, gluconate, or fumarate) providing 60 mg of elemental iron daily for mild anemia and 120 mg plus 0.4 mg folic acid daily for moderate or severe anemia treatment should continue for 4–6 weeks past normalization of hemoglobin.^83^ Adding ascorbic acid increases iron absorption. Intravenous iron infusions in pregnant women with iron deficiency anemia result in greater hemoglobin increases compared with oral iron supplements,^68^ but the cost of this intervention may limit feasibility in lower-resource settings.^84^

Other nutritional causes of anemia include deficiencies of vitamin B12, folate, and vitamin C. Macrocytic anemia may be due to vitamin B12 and/or folate deficiency. If further assessment to enable differential diagnosis is not available, treatment should always include vitamin B12. The recommended course is 1 mg of vitamin B12 administered intramuscularly 2–4 times per week until hematological parameters return to normal, then continued monthly.^83^ Folate deficiency is addressed with oral supplementation of 0.5 mg folic acid daily for 4 months, or until term if treatment is occurring during pregnancy.^83^ Treatment of vitamin C deficiency requires daily oral supplementation with a tablet containing 500 mg vitamin C plus folic acid.^83^

Uncomplicated *P. falciparum* malaria in children and adults should be treated with a 3-day course of artemisinin-combination therapy (ACT); dihydroartemisinin + piperaquine is recommended for general use, but there are four other WHO-recommended ACT formulations.^48^ The exception is pregnant women in the first trimester, for whom the recommended treatment is 7 days of quinine + clindamycin.^48^ To prevent relapse, the WHO recommends treating infection with *P. vivax* or *P. ovale* in children and adults (except individuals with G6PD deficiency, pregnant women, infants below 6 months of age, women breastfeeding infants below 6 months of age, or older infants unless they are known not to be G6PD deficient) with a 14-day course of primaquine at 0.25–0.5 mg/kg body weight daily in all transmission settings.^48^

Individual treatment for helminth infection involves a single 400 mg dose of albendazole, or mebendazole 500 mg orally once or 100 mg orally twice a day for 3 days.^83^ Schistosomiasis infection should be treated with praziquantal.^62^

Blood transfusions are necessary in cases of acute hemorrhage leading to shock, to restore oxygenation of the tissues.^83^ For children with severe anemia, transfusion within 24 h is indicated if hemoglobin is below 4 g/dL or 4–6 g/dL with relevant clinical symptoms (i.e., respiratory distress, impaired consciousness, etc.), with additional considerations in cases of severe acute malnutrition.^85^ If available, packed cells (10 mL/kg) are preferred to whole blood (20 mL/kg); either blood product should be given over 3–4 h.^85^ Transfusion is also indicated for severe anemia in individuals with sickle cell disease. In addition, some thalassemias are transfusion-dependent and require iron chelation therapy as well as daily oral supplementation with 500 mg vitamin C and 5 mg folic acid.^83^

## MAJOR GAPS OR CHALLENGES IN GENERATING EFFECTIVE ACTION AND IMPACT

### Gaps specific to key WHO-recommended interventions

#### Supplementation

- Current iron supplementation guidelines are based on meta-analyses of studies in which iron formulations, dosages, and intervention durations varied widely. The reviews identified for this paper did not report comparisons between iron formulations for either efficacy or safety. Research on the efficacy and safety of different types of iron formulations could lead to more specificity in future guidelines on iron supplementation.
- Trials or rigorous effectiveness evaluations assessing the acceptability and adverse effects of interventions based on the WHO iron supplementation guidelines are lacking.
- Evidence of the effectiveness of iron supplementation for reducing anemia in children 24–59 months and in postpartum women is currently lacking.
- There is a knowledge gap regarding the effects of daily iron supplementation on nonhematological outcomes, including children’s growth and cognitive development.
- For women of reproductive age, evidence for clinically relevant outcomes beyond anemia and iron status, including fatigue, cognitive performance, psychological health, and productivity, are lacking, as well as assessment of the effects of preconception iron supplementation on pregnancy and birth outcomes.
- There are knowledge gaps on the optimal combination of micronutrients, formulations, doses, and duration of supplement use for each life stage for both the prevention and treatment of anemia.

#### Fortification

- The use of the most bioavailable forms of micronutrients and ensuring industry adherence and compliance to fortification standards through strong quality control mechanisms will increase the effectiveness of staple food fortification initiatives. Evidence is lacking on the bioavailability of different iron compounds for fortifying wheat, maize, and rice, as well as the bioavailability and stability of vitamin A for fortification of different grains with the use of various processing techniques.^40,42,44^
- For wheat and rice fortification, there are gaps in understanding the effect of phytate on the bioavailability of different forms of iron and methods of mitigating this effect.^40,44^
- For rice fortification, evidence limitations include the bioavailable forms of iron that do not change the color of the rice grain, understanding micronutrient stability with different cooking processes, and validation of assays for the assessment of vitamin and mineral content of fortified rice.^44^
- For maize flour and corn meal, knowledge gaps persist regarding the appropriate fortificant levels, stability of micronutrient compounds under different processing methods, and the physical properties of fortified products and their acceptability to consumers.^42^

#### Malaria prevention

- Limitations exist regarding the evidence for the impact of IPT for malaria prevention in pregnant women and school-aged children aged 5–15 years.
- The inclusion of anemia as an outcome is lacking in malaria programs and in implementation research for malaria programs.

#### Helminth control

- Although the current evidence suggests that preventive anthelmintics provided to pregnant women may reduce maternal anemia, previously it indicated no effect, and the certainty of the current evidence is low, especially regarding the impact that different drugs have on anemia.
- Evidence is lacking on the effects on anemia of mass deworming campaigns in adolescent girls and women of reproductive age from areas that are endemic for soil-transmitted helminths.
- Evidence is lacking on the effect of preventive chemotherapy for schistosomiasis on anemia for different population groups.

#### Cross-cutting

- Given the complex etiology of anemia, multisectoral interventions likely need to be implemented synchronously to address the priority determinants in specific contexts. However, most of the evidence base is from studies of single interventions, with limited data on the potential synergies and risks of more comprehensive programs.^15^ This is a critical gap and could be addressed by modeling and evaluation of the effects of integrated programs.
- There is a lack of evidence regarding multidrug and drug–nutrient interactions in comprehensive anemia programming, particularly in the context of concurrent initiatives to address malaria, helminths, and micronutrient status. Applying these insights in conjunction with national data on key drivers of anemia would assist in the determination of an appropriate balance between iron- and infection-related interventions in different settings.
- There are also gaps in evidence regarding effective interventions to prevent and control anemia through the reduction of inflammation or the management of genetic blood disorders.
- Assessment of iron intake from all sources could help accurately determine safe and effective dosing regimens and to guide the optimization of fortification and supplementation protocols in the context of concurrent implementation.
- Many studies of interventions addressing anemia determinants other than iron deficiency do not assess anemia as an outcome and thus were excluded from the literature reviewed for this paper.
- The WHO guidelines for interventions such as vitamin A supplementation and control of helminths and malaria focus on the relevant primary outcome and generally do not also consider the evidence that does exist for effects on hemoglobin and anemia. Two potential limitations of this omission are that the recommended dosing regimens may not be optimized for anemia prevention and control and that these interventions may be excluded from national anemia strategies. The latter may be mitigated by the inclusion of guidelines on anemia prevention and control interventions for multiple determinants in the WHO’s nutritional anemia tool.^82^

## WAYS FORWARD AND INNOVATION NEEDED

Individual interventions for anemia prevention and control must be understood as elements of a larger program interacting with the complex determinants of anemia and comorbidities in a given context. This will shape both the interpretation of effectiveness data and the selection of interventions for inclusion or strengthening within country programs, as well as guiding future research priorities. As highlighted in the Gaps section above, there is a lack of evidence regarding the optimization of intervention protocols through improved understanding of intake adherence, adverse effects, and interactions between drugs and nutrients. A better understanding of what is an optimal mix of interventions implemented in a country, maximizing impact on nutrition and health outcomes while augmenting the cost−benefit of the investment and minimizing unwanted risks, could also result in effective anemia reduction programs. Technological innovations could improve the effectiveness of food fortification programs. This is particularly true for rice fortification and DFS, where organoleptic issues have been reported.^86,87^

Anemia has multiple determinants but has been historically linked most closely with iron deficiency, limiting the available data on the effectiveness of interventions addressing other relevant micronutrient deficiencies, infections, or other causes. Thus, the evidence base could be strengthened by including anemia indicators in trials of interventions addressing determinants other than iron deficiency, and the resulting evidence regarding safe and efficacious dosing regimens for anemia prevention and control could be included in global guidelines. This will assist countries in including and prioritizing these interventions, where appropriate, in national anemia strategies. There is a particular need for investigation of the potential to reduce anemia prevalence through the prevention and control of inflammation, and to be able to better detect and manage inherited red blood cell disorders.^4^

Implementation research could identify and strengthen delivery platforms for specific groups in different settings. This could improve the quality, coverage, and acceptability of anemia intervention delivery and result in determining the best way to package the delivery of multiple interventions.^88,89^ This is required for both long-standing programs such as IFA for pregnant women, which continues to be hampered by low coverage rates,^89^ and for newer interventions such as rice fortification. Identifying appropriate delivery platforms to reach specific populations with fortified foods is particularly challenging in rural areas reliant on local agriculture, bypassing large-scale processing. The feasibility of implementing and regulating small- and medium-scale fortification on a sustainable basis has yet to be demonstrated but continues to hold potential.^90^ Further research on the effectiveness, feasibility, and acceptability of biofortification is also needed, as changes in farming methods may be required, as well as consumer acceptance of a change in the color or taste of familiar staple foods.^90^

## CONCLUSIONS

In summary, several WHO-recommended interventions are effective in preventing anemia, primarily through increasing iron intake through supplementation and fortification, and decreasing infections (e.g., malaria). There is a lack of evidence on the effect of deworming programs on anemia as the current quality of the evidence is low.

- Daily iron supplementation reduces the risk of anemia in children aged 6–23 months, children aged 5–12 years of age, menstruating adult women and adolescent girls, and pregnant women but there is insufficient evidence available regarding the effect on anemia among children 2–5 years of age.
- Intermittent iron supplementation (once or twice weekly) is as effective as daily supplementation in preventing anemia among menstruating girls and women.
- MNP decreases the risk of anemia in children 6–23 months and in preschool and school-aged children.
- Fortification of wheat flour with iron, with or without other micronutrients, reduces the risk of anemia in the general population above 2 years of age, and currently, it is uncertain whether fortification of maize flour or rice with iron reduces the risk for anemia in the general population. Fortification with multiple micronutrients decreases the risk of anemia.
- IPT with SP may reduce the risk of anemia in pregnant women and in school-aged children aged 5–15 years. IPT likely decreases the risk of anemia in infants. SMC likely reduces the prevalence of anemia at the end of the malaria transmission season in children under 5 years of age.
- A single dose of anthelmintics in the second trimester of pregnancy may decrease maternal anemia in the third trimester.
- Mass deworming campaigns for soil-transmitted helminths in children aged 6 months to 16 years of age and in adolescent girls and women of reproductive age do not seem to impact hemoglobin concentrations. Multiple doses of deworming drugs administered to children 6 months to 16 years of age likely do not decrease anemia.

Promising interventions to prevent anemia include vitamin A supplementation, MMS, LNS, DFS, and biofortification. Some studies have shown that vitamin A supplementation can reduce the risk of anemia in children, adolescents, and pregnant women. MMS has been shown to be equally effective as IFA in reducing the risk of anemia in the third trimester of pregnancy. LNS plus complementary feeding may reduce anemia in children 6–23 months of age, while DFS may reduce the risk of anemia in the general population. Consumption of iron-biofortified crops does not appear to decrease the risk of anemia in people aged 12–45 years, but the evidence to date is very limited.

Additional evidence to optimize the effectiveness of interventions could include identifying the most bioavailable forms of iron for supplementation and fortification, examining adherence to and acceptability of all potential interventions, and identifying the optimal deworming and IPT dosage and timing to address anemia. Technical guidance exists to inform the country-level design, implementation, and scaling-up of nutrition programs for combating nutritional anemias that can be tailored based on national studies on the etiology and distribution of anemia.

## Figures and Tables

**TABLE 1:** Effect of interventions on anemia.

Author (Reference)	Year	Intervention	Comparison	Population	Number of studies	Outcome	Effect	Quality of the evidence
*WHO recommended interventions*								
*Supplementation*								
Manapurath et al.^10^	2022	Enteral iron supplementation	Control	Infants born preterm or low birth weight	2	Anemia	Positive RR: 0.25 (0.10, 0.62)	Moderate
Pasricha et al.^13^	2013	Daily iron supplementation	Control or cointervention without iron	Children 4–23 months	17	Anemia	Positive RR: 0.61 (0.50, 0.74)	Not specified
Low et al.^12^	2013	Daily iron supplementation	Control	Children 5–12 years	7	Anemia	Positive RR: 0.50 (0.39, 0.64)	Not specified
Thompson et al.^14^	2013	Daily iron supplementation	Control or cointervention without iron	Children 2–5 years	1	Anemia	N/A	Not specified
Low et al.^22^	2016	Daily iron supplementation	Control	Nonpregnant women 12–50 years	10	Anemia	Positive RR: 0.39 (0.25, 0.60)	Moderate
Fernández-Gaxiola et al.^23^	2019	Intermittent iron supplementation	Control or placebo	Nonpregnant women 12–50 years	11	Anemia	Positive RR: 0.65 (0.49, 0.87)	Low
Pena-Rosas et al.^26^	2015	Daily iron supplementation	Control/placebo	Pregnant women	14	Anemia	Positive RR: 0.30 (0.19, 0.46)	Low
					9	Severe anemia	Unclear RR: 0.22 (0.01, 3.20)	Very low
Pena-Rosas et al.^25^	2015	Intermittent iron supplementation	Daily iron supplementation	Pregnant women	4	Anemia	Unclear RR: 1.22 (0.84, 1.80)	Very low
					6	Severe anemia	N/A	Very low
Moorthy et al.^15^	2020	Daily iron supplementation	Control	Children 6–59 months	29	Anemia	Positive RR: 0.59 (0.5, 0.69)	Not specified
WHO^28^	2016	Daily iron supplementation	Control	Postpartum women	1	Anemia	Positive RR: 0.35 (0.18, 0.65)	Low
*Point-of-use fortification of foods with multiple micronutrient powders (MNPs)*								
Suchdev et al.^38^	2020	Home fortification with MNP	No intervention or placebo	Children 6–23 months	16	Anemia	Positive RR: 0.82 (0.76, 0.90)	Moderate
De-Regil et al.^39^	2017	Home fortification with MNP	No intervention or placebo	Children 24 months to 12 years	10	Anemia	Positive RR: 0.66 (0.49, 0.88)	Moderate
*Fortification*								
Field et al.^41^	2021	Wheat flour fortified with iron with or without other micronutrients	Wheat flour (no added iron) with or without other micronutrients	General population older than 2 years of age	5	Anemia	Positive RR: 0.73 (0.55, 0.97)	Low
		Wheat flour fortified with iron with or without other micronutrients	Unfortified wheat flour	General population older than 2 years of age	2	Anemia	Unclear RR 0.77 (0.41, 1.46)	Very low
Garcia-Casal et al.^43^	2018	Maize flour or maize flour products fortified with iron plus other vitamins and minerals	Unfortified maize flour or maize flour products	General population older than 2 years of age	2	Anemia	Unclear RR: 0.90 (0.58, 1.40)	Very low
Peña-Rosas et al.^45^	2019	Rice fortified with iron alone or in combination with other micronutrients	Unfortified rice	General population older than 2 years of age	7	Anemia	Positive RR: 0.72 (0.54, 0.97)	Low
Das et al.^46^	2019	Multimicronutrient fortification of various foods	Placebo or no intervention	General population	11	Anemia	Positive RR: 0.68 (0.56, 0.84)	Low
*Malaria prevention*								
WHO^48^	2022	Intermittent preventive treatment (sulfadoxine-pyrimethamine)	Control	Pregnant women	53	Anemia	Positive RR: 0.90 (0.87, 0.93)	Low
Esu et al.^49^	2021	Perennial malaria chemoprevention (sulfadoxine-pyrimethamine)	Placebo or Control	Children 1–12 months	6	Anemia	Positive RR: 0.82 (0.68, 0.98)	Moderate
Cohee et al.^50^	2020	Intermittent preventive treatment	Control	School-aged children 5–15 years	12	Anemia	Positive ARR: 0.85 (0.77, 0.92)	Low
WHO^48^	2022	Seasonal antimalarial chemoprevention	Control	Children under 5 years	6	Anemia	Positive RR: 0.84 (0.80, 0.88)	Moderate
				Children 5–9 years	1	Anemia	Positive RR: 0.70 (0.52, 0.95)	High
		Postdischarge malaria chemoprevention	Placebo	Children under 10 years	3	Readmission for severe anemia	Unclear HR: 0.74 (0.52, 1.05)	Moderate
Pryce et al.^53^	2018	Insecticide-treated bednets	Control	People of all ages	5	Anemia (mean packed cell volume)	Positive MD: 1.29 (0.42, 2.16)	High
*Deworming*								
Salam et al.^61^	2021	Anthelmintics	Placebo or Control	Pregnant women in second trimester of pregnancy	5	Anemia in third trimester	Positive RR: 0.85 (0.72, 1.00)	Low
Taylor-Robinson et al.^58^	2019	Anthelmintics–single dose	Placebo or Control	School age children in areas endemic for soil-transmitted helminths	3	Hemoglobin	No effect MD: 0.06 g/dL (−0.05 lower–0.17 higher)	Moderate
		Anthelmintics–multiple doses			7		No effect MD: 0.02 g/dL lower (0.08 g/dL lower–0.04 g/dL higher)	Low
Tanjong Ghogomu et al.^59^	2018	Anthelmintics	Placebo or Control	Adolescent girls and women of reproductive age from areas endemic for soil-transmitted helminths	3	Anemia	Unclear RR 0.82 (0.60, 1.11)	Low
					1	Iron deficiency anemia	Unclear RR 0.89 (0.64, 1.23), 1 study	Low
Byrne et al.^60^	2021	Albendazole	Control	Nonpregnant populations (preschool and school age children, adult males, adult nonpregnant females)	10	Hemoglobin	Positive SMD: 0.21 (0.12, 0.29)	Not specified
		Albendazole + praziquantal			10		Positive SMD: 0.27 (0.18, 0.36)	
		Mebendazole			6		No effect SMD: 0.04 (−0.08, 015)	
*Prevention and treatment of postpartum hemorrhage with uterotonics*								
Salati et al.^65^	2019	Oxytocin	Placebo or Control	Birthing women with vaginal deliveries	2	Severe anemia 24–48 h postpartum	Unclear RR: 0.64 (0.18, 2.26)	Moderate
*Management of menstrual blood loss with hormonal contraception*								
Lethaby et al.^67^	2019	Combined oral contraceptive pills	Placebo	Women with heavy menstrual bleeding	2	Hemoglobin	Positive but meta-analysis not performed	Not specified
*Potentially promising interventions*								
*Vitamin A supplementation*								
McCauley et al.^71^	2015	Vitamin A supplement	Placebo or Control	Pregnant women	3	Anemia	Positive RR: 0.64 (0.43, 0.94)	Moderate
				Neonates	1	Anemia	No effect RR: 0.99 (0.92, 1.08)	High
da Cunha et al.^69^	2019	Vitamin A supplement	Placebo (RCT) or baseline (pre-post evaluation)	Children and teenagers 1–19 years	9	Anemia	Positive RR: 0.72 (0.64, 0.82)	Not specified
				Pregnant and lactating women 17–35 years	1	Anemia	Positive RR: 0.78 (0.63, 0.96)	Not specified
*Multiple micronutrient supplements (MMSs)*								
WHO^70^	2020	MMS	Iron and folic acid supplement	Pregnant women	8	Anemia	Equally effective RR: 1.03 (0.92, 1.15)	High
Keats et al.^91^	2019	MMS	Iron supplementation with or without folic acid	Pregnant women	9	Anemia	Equally effective RR: 1.04 (0.94, 1.15)	Not specified
		MMS	Placebo	Pregnant women	1	Anemia	Positive RR: 0.66 (0.51, 0.85)	Not specified
Keats et al.^92^	2022	MMS	Iron and folic acid supplement	Adolescents 10–19 years	8	Anemia	Equally effective RR: 1.07 (0.86, 1.33)	Not specified
*Lipid nutrient supplements (LNSs)*								
Das et al.^72^	2019	LNS plus complementary feeding	Placebo or Control	Children aged 6–23 months	5	Anemia	Positive RR: 0.79 (0.69, 0.90)	Low
		LNS plus complementary feeding	Multiple micronutrient powders	Children 6–23 months of age	2	Anemia	Positive RR: 0.38 (0.21, 0.68)	Low
Das et al.^74^	2018	Iron and folic acid supplement	LNS	Pregnant women	1	Anemia	Negative RR: 2.35 (1.67, 3.3)	Moderate
		Multiple micronutrient supplement	LNS	Pregnant women	1	Anemia	Negative RR: 1.4 (1.07, 1.82)	Moderate
Dewey et al.^73^	2021	Small quantity LNS	Control	Children 6–24 months of age	14	Anemia	Positive Relative Reduction: 16% (13, 19)	Not specified
*Double-fortified salt with iodine and iron (DFS)*								
Larson et al.^77^	2021	DFS	No intervention or iodized salt	Children, adolescents, and women	13	Anemia	Positive RR: 0.80 (0.70, 0.92)	Not specified
Ramírez-Luzuriaga et al.^78^	2018	DFS	Iodized salt	General population	8	Anemia	Positive RR; 0.59 (0.46, 0.77)	Not specified
Baxter et al.^76^	2022	DFS	Iodized salt	General population	8	Anemia	Positive RR: 0.79 (0.66, 0.94)	Moderate
*Biofortification*								
Finkelstein et al.^80^	2019	Iron-biofortified crops (rice, pearl millet, and beans)	Conventional crops	General population	3	Anemia	Unclear OR: 0.83 (0.58, 1.19)	Not specified

Abbreviations: ARR, adjusted relative risk; DFS, double-fortified salt; HR, hazards ratio; IFA, iron and folic acid supplement; IPT, intermittent preventive treatment; LNS, lipid-based nutrient supplement; MMS, multiple micronutrient supplement; MNP, multiple micronutrient powder; OR, odds ratio; RCT, randomized controlled trial; RR, relative risk; SP, sulfadoxine-pyrimethamine.
